# New Strategies for Evaluation and Analysis of SELEX Experiments

**DOI:** 10.1155/2014/849743

**Published:** 2014-03-19

**Authors:** Rico Beier, Elke Boschke, Dirk Labudde

**Affiliations:** ^1^Bioinformatics Group, Department of Mathematics, Natural and Computer Sciences, University of Applied Sciences Mittweida, 09648 Mittweida, Germany; ^2^Institute of Food Technology and Bioprocess Engineering, Department of Mechanical Engineering, Dresden University of Technology, 01062 Dresden, Germany

## Abstract

Aptamers are an interesting alternative to antibodies in pharmaceutics and biosensorics, because they are able to bind to a multitude of possible target molecules with high affinity. Therefore the process of finding such aptamers, which is commonly a SELEX screening process, becomes crucial. The standard SELEX procedure schedules the validation of certain found aptamers via binding experiments, which is not leading to any detailed specification of the aptamer enrichment during the screening. For the purpose of advanced analysis of the accrued enrichment within the SELEX library we used sequence information gathered by next generation sequencing techniques in addition to the standard SELEX procedure. As sequence motifs are one possibility of enrichment description, the need of finding those recurring sequence motifs corresponding to substructures within the aptamers, which are characteristically fitted to specific binding sites of the target, arises. In this paper a motif search algorithm is presented, which helps to describe the aptamers enrichment in more detail. The extensive characterization of target and binding aptamers may later reveal a functional connection between these molecules, which can be modeled and used to optimize future SELEX runs in case of the generation of target-specific starting libraries.

## 1. Introduction

The inhibition of protein interactions, such as receptor-ligand interactions or the interplay during pathogen infections, is one main functional principle of therapeutics to influence biologically relevant processes. In this context usually antibodies are used to bind to specific target proteins and thus wield biological influence. Although antibodies and corresponding technologies are widely distributed, they are accompanied with some major drawbacks. A first hindrance is the antibody's large size that limits the access to smaller biological compartments and thus also its bioavailability. It is also problematic that antibodies are often immunogenic and cannot be used after their denaturation. If we consider the production process of antibodies, it becomes apparent that this process is difficult to scale up and susceptible to bacterial or viral contamination [1, 2]. The need of finding other target-binding molecules as alternatives for antibodies draws the attention now to another surrogate, the aptamer, which is also qualified for target binding [1].

These aptamers are short and stable, single-stranded nucleotide oligomers folding into complex three-dimensional structures. They are composed of helical parts and different variants of loops like hairpins, inner loops, bulges, and junctions, which allow branching of the structure. Unpaired nucleotides have a higher potential to take part in intermolecular, noncovalent chemical bonding via hydrogen bonds, hydrophobic, and electrostatic interactions on the nucleotides preferred binding sites [3]. Aptamers can target a diverse multitude of particles from small molecules like organic dyes [4] and amino acids [5] and larger molecules like antibiotics [6] and proteins [7] as well as whole cell surfaces [8]. The focus on therapeutically applied aptamers lies especially on proteins as target molecules. Notably, in respect of binding affinity they are comparable to antibodies. While a study has shown that an aptamer with an affinity of *K*_*d*_ = 50 pM could be found for vascular endothelial growth factor as target, an antibody for the same target in comparison shows an affinity of *K*_*d*_ = 54 pM [9, 10]. Furthermore there is growing evidence of a connection between regions of unpaired nucleotides and the concrete biological function of RNA molecules. This can analogously be assumed for DNA aptamers [11].

Since the production process of aptamers is purely chemical, it is readily scalable and less prone to bacterial or viral contamination, which poses an advantage over artificial synthesis of antibodies [1, 2]. The resulting aptamers are usually not immunogenic and smaller in size, which allows a less elaborate administration of aptamer based medication [12]. Although the aptamer denaturation is reversible, their half-life is limited by nuclease degradation. This vulnerability can only be opposed by chemical modification of the aptamers [1]. In summary, aptamers are an attractive alternative to antibodies and will lead to new issues in the fields of bioinformatics.

With the introduction of next generation sequencing (NGS) technologies it is possible to massively parallelize the sequencing process. That makes it easy to gather large amounts of sequence data in relatively short periods of time [13]. In this manner the NGS technology can be used for genome sequencing to speed up and enhance the shotgun sequencing. But that is not the only use of NGS. The sequencing technology is also applicable in fields of aptamer research, especially in the process of finding high affinity aptamers for a desired target molecule. Caused by the high complexity of the conformational space of aptamers it is a hard problem to find target-binding aptamers. Commonly a screening technology needs to be utilized to find these unique aptamers that are capable of binding to a specific target molecule. This technique is called SELEX (Systematic Evolution of Ligands by Exponential Enrichment) [14]. During the multiple steps of the experimental process there are several opportunities for performing NGS to gather sequence data useful for the purpose of later analysis.

The SELEX screening process starts with a chemically synthesized, random library of nucleotide oligomers of a fixed size. Although the size of this starting library is fairly large with a range of typically 10^13^ to 10^16^, it can in practice only cover a small fraction of the possible sequence and structure space, because these spaces are growing exponentially with the desired aptamers lengths. Based on this library multiple subsequent selection rounds are performed, in which library and target molecules are incubated. As the multitude of aptamers contained in a rounds library is competing for the fewer binding sites available on the relatively small number of target molecules added, the arising selection pressure leads to the preferred binding of the highest affinity oligonucleotides of the library. Commonly some experimental parameters are adjusted during the execution to increase this selection pressure during the incubation. After each SELEX iteration nonbinding candidates are washed out and the bound aptamers are prepared for the next round. This includes the elution of aptamer candidates from target molecules and a following amplification to obtain a library sufficient in size for the next round. Only oligonucleotides capable of binding to the target or background materials necessary for carrying out the experiment are enriched during that process [14]. This leads to the enrichment of specific and affine aptamers and thus a decrease of diversity in the resulting library can be observed.

NGS techniques now provide the possibility to better analyze such SELEX experiments. Benefits are provided by the magnitudes of higher sequencing coverage of the real library sequence diversity compared to classic sequencing technologies, such as Sanger sequencing [15], and the possibility to gain information from all SELEX rounds with reasonable effort. Hence, it is no longer only the final round that can be analyzed, but rather the development of the library during the whole experiment, which provides new chances in bioinformatics analysis. Nevertheless, the next generation sequencing technology is despite its advantages accompanied by some major drawbacks. NGS is a high throughput sequencing technique, which means that one has to consider sequencing errors. Although the probability of each single base being sequenced incorrectly is quite low, denoted by Phred values up to 41, the large number of single base reads within each data set will induce many sequencing errors [16]. Another problem is that the limits of conventional algorithms and their implementations can easily be reached when processing large NGS data sets.

If one is able to handle these difficulties, the additional information source provided by the NGS technology when performing SELEX experiments allows a deeper analysis and understanding of the SELEX process. So the analysis of only the first rounds of a SELEX experiment may show specific enrichment of the library and thus draws a deduction towards the enrichment of the final round. This could be a first hint for sequence characteristics that yield target-specific binding affinity. Those observations would allow interrupting a running SELEX experiment, skipping some intermediate selection rounds, and instead continuing with a computationally enriched pool at later position, saving time and material expenses. The enrichment of the aptamer library during the SELEX process can be observed when analyzing the sequence data gained from the different rounds. Using the NGS data, a diversity indicator can be calculated and compared, showing that the number of different sequences effectively decreases. It is very important to find a proper description for the observed aptamer enrichment in the later SELEX rounds. Though the simple description of the enrichment as a list of most frequently observed aptamers in the data set is sufficient for conventional validation of the experiments success through concrete binding experiments, a better way of description has to be found when aiming at the improvement of prospective SELEX runs.

The enrichment has to be characterized and more detailed, because occurring commonalities between the different found aptamer sequences indicate characteristics of the aptamers at different physical positions, which are relevant for binding to the target. Sequence motifs are one opportunity to describe those shared features on sequence level. These motifs are in turn corresponding to substructures within the aptamers, which are characteristically fitted to specific binding sites located on the target molecules surface and therefore are present in all binding aptamers. Using a position specific scoring matrix as motif representation allows the definition of variable regions, which better reflects the natural divergence and thus preserves the informational content gained from the NGS sequence data. Once found, the sequence motifs can be utilized to generate an enriched and thus improved and target-specific starting library for SELEX experiments, which will positively affect the progress of future SELEX runs on the same target molecule. This would imply that for each improved SELEX run another experiment has to be performed to gain the information needed for generating the target-specific starting library for the main experiment. The real practical benefit of the motif description of the sequence libraries enrichment during the SELEX experiments becomes apparent, when later using the motifs as descriptors for the target molecule. The effect can be extended by using multiple bioinformatics technologies, ranging from sequence analysis by employing sequence alignment strategies and clustering techniques to secondary and tertiary structure prediction as well as the aforementioned motif search. Other technologies like electrostatic calculation and docking simulation are utilizing concrete three-dimensional structure information, which can be acquired from databases, through own structural clarification or structure prediction. Combining all these techniques it will be possible to extract a set of descriptors for both, target molecule and found aptamers, which characterize the aptamer-target-binding. These descriptors now need to be correlated appropriately to build an abstract model describing the aptamer-target-binding relation. The model can then be applied to an unknown target molecule in an effort to obtain information on the composition and architecture of binding aptamers only based on information about the desired target. The generation of target-specific SELEX starting libraries without the need of concrete performed previous experiments with the desired target would greatly improve the aptamer finding process.

This paper will present a search technique using suffix trees to find recurring motifs in large NGS nucleotide sequence data sets as one methodology besides the other mentioned techniques allowing deriving target-related descriptors for the later generation of target-specific SELEX starting libraries. This method is exemplarily attempted on an NGS data set supplied from a SELEX experiment targeting a* Norovirus* capsid protein.

## 2. Data Set and Investigated Target

In the past a SELEX experiment was performed to find a DNA aptamer capable of binding to the* Norovirus* genotype II.4 capsid protein VP1 as its target [17]. This aptamer may be used for efficient* Norovirus* detection or infection control. For validation of the successful enrichment of sequences during the experiment and further analysis profiting from the much higher coverage, next generation sequencing was performed to gather sequence data for all screening rounds.

### 2.1. Target

The* Norovirus* has been detected in 1972 in Norwalk, USA, for the first time. Since then this virus could be found in a variety of different genotypes spread all over the world. The* Norovirus* belongs to the family Caliciviridae and is genetically diverse.* Noroviruses* are the major cause of viral epidemic gastroenteritis worldwide, often resulting in large and persisting outbreaks. Two of the five major genogroups, GI and GII, especially the genotype GII.4, are responsible for the majority of human infections. Since only few viruses are already able to cause an infection, they are highly contagious. To the present there is no vaccine available, which could prevent a* Norovirus* disease outbreak [18]. The* Norovirus* contains a single-stranded, positive-sensed RNA genome with an approximate size of 7.7 kb, which is enclosed in a nonenveloped protein coat. This coat exhibits distinct cup-shaped depressions. Its icosahedral capsid structure is formed by 90 dimers of the capsid viral protein 1 (VP1), which is assembled of two domains. The inner S domains form a shell around the RNA, whereas the P domains are protruding on top of the shell [19]. Another minor capsid protein (VP2) is only present in a few copies. The overall construct leads to thermal stability of the virus, allowing it to survive temperatures up to 55°C and a pH in the range of 3–7 [20].

At present, a* Norovirus* infection is usually diagnosed by reverse transcription PCR (RT-PCR) or enzyme-linked immunosorbent assay (ELISA) using anti-*Norovirus* antibodies. Although the cost-intensive RT-PCR is the most sensitive method known so far, the genetic diversity of* Noroviruses* does not allow testing for all genotypes in one assay. Attributable to their low sensitivity ELISA assays can only be used for screening, where the results are confirmed by a following RT-PCR [21]. In a recent development an immunochromatographic detection assay based on antibodies was rated to have a high sensitivity and specificity [22]. As there is still a strong need for point-of-care methods for* Norovirus* detection, a solution using aptamers as receptor units may be another chance to develop real-time, label-free, and possibly low-cost biosensor systems for* Norovirus* detection. Targeting the attachment and internalization of the virus, one interesting approach would be to inhibit the binding of the P2 subdomain to its receptor molecules by competitive interacting molecules. Hence,* Norovirus* binding aptamers might also be used in vivo to control* Norovirus* infection.

### 2.2. Origin of Sequence Data

The target capsid protein VP1 of* Norovirus* genotype II.4 was expressed as a recombinant with polyhistidine-tag appended for later immobilization. The sequences of the initial library contained a 49 nt long random section enclosed by the necessary primers. So the initial library is described by the following template sequence: 5′ GCC TCT TGT GAG CCT CCT AAC -N_49_- CAT GCT TAT TCT TGT CTC CC 3′. The SELEX experiment was performed in twelve rounds. After every third selection round an additional negative selection was performed to remove aptamer candidates binding to background materials of the experiment or to fecal specimen, the later sample matrix.

For each round of the SELEX experiment the next generation sequencing supplied a sequencing file in FASTQ format containing the aptamer sequences remaining after this round. For each sequenced base the file further contains an additional coded quality value which approximates the error probability at this position. The sequences are flanked by parts of the Illumina primer sequences.

### 2.3. Preparation of Sequence Data

Prior to any concrete sequence analysis a preprocessing step of the raw data produced by the sequencer needs to be done. The aptamer sequences are flanked by primer sequences. At first these primer sequences, either fully preserved or just fragments, have to be recognized and removed. Raw sequences that did not contain the given primer sequences have been rejected. The remaining inserts are the object of the intended motif search.

Each sequenced base is annotated with a coded quality value which approximates the error probability at this position. Although these quality values are not regarded as an absolute quality indicator, conspicuously low values or continuous sections exhibiting low values may indicate sequencing errors. As the main goal of a SELEX experiment is the enrichment of the sequence pool with binding aptamers, a sequence occurring only with very small quantity can also be considered as deficient. Based on this information a filter can be applied, which discards sequences of possibly low quality. After preparation the data set contained approximately 233000 sequences, from which 5500 sequences were distinct.

## 3. Motif Search

As intended, this study is aimed at developing a search technique using suffix trees to find recurring sequence motifs, which are corresponding to concrete binding areas of the aptamers. The prepared sequence data of the SELEX experiment described above is the basis for the following search strategy, which will be presented in three main steps. After a short overview of different approaches of motif search utilizing suffix trees, the generation of a generalized suffix tree, which is used by a later exhaustive search, is described. Here, also the possibility of using only subsequences located on loop regions of the predicted structures is mentioned. Thereafter the benefit of the tree structure in doing a full search is outlined. The last part explains a couple of termination criteria for the search. Afterwards a possible way to handle the results of a motif search easier is specified.

### 3.1. Suffix Tree Based Motif Search

Over the last three decades suffix trees have been repeatedly utilized for sequence matching as they are known to provide very fast string operations [23]. The most simplistic problem is to find the exact motifs occurring in a subset of the given sequences. In particular, this can be done by traversing the tree to find nodes visited by the denoted minimum number of sequences. This basic problem increases in complexity when more meaningful biological demands are considered. This includes the incorporation of character mismatches and sequence gaps during computation. With respect to DNA sequences and their corresponding structures, single motif elements can interact spatially and may be important for structure stabilization or even for defining the three-dimensional fold. However, such motif elements are not necessarily located in direct sequence neighborhood, which requires considering long gaps between elements. A number of approaches target the finding of such gap-containing motifs. Early algorithms permitted only fixed gap lengths—a restriction, which limits the number of possible motif arrangements. More sophisticated algorithms are also able to handle motifs interrupted by gaps of variable lengths [24–26]. In addition, integrating sequence-specific biological relevance to the problem of pattern and motif identification requires an appropriate ranking and processing scheme [27]. The other aspect of this problem lies in defining mismatch acceptance within motif hits, which would allow regarding mutations occurring in evolutionary processes, such as SELEX. These algorithms are usually intended to find motifs containing up to a fixed number of mismatches within each occurrence. In most cases, the mismatches are not restricted by special rules [28, 29]. The aforementioned algorithms directly aim at finding motifs within the suffix tree. An alternate approach affords ranking the search space to find a subspace (subtree) containing appropriate motif hits [30].

Brazma et al. introduced the Pattern Discovery Algorithm, which realizes an exhaustive search for three different classes of motifs. One of these classes called “patterns with character groups” describes mismatches by means of a well-defined regular expression syntax, which helps to specify the motif variability more precisely. The algorithm uses a suffix tree where nodes are annotated with symbols of the employed regular expression syntax, which means the character groups. This massively increases the tree size and thereby limits its practice [31]. Following the example of an exhaustive search over all possible patterns including variable regions within a huge number of nucleotide sequences, the single string search for one pattern in a single sequence needs to be optimized in order to minimize computational costs. In contrast to the Pattern Discovery Algorithm, our approach uses the generalized suffix tree annotated with the letters of the sequence alphabet. The consideration of variability is realized by merging nodes during the later search phase, which reduces memory usage and avoids the creation of unnecessary subtrees that would be created in the character group based tree. In this study, biological relevance is derived from predicted secondary structure information. In particular, free energy estimations of predicted structures are employed to ranking corresponding sequences prior to motif search, which, to our knowledge, poses a novelty in this field.

### 3.2. Tree Construction

To project sequences onto this tree structure, each edge of the generalized suffix tree is annotated with one of the possible characters of the underlying alphabet. Internally each character is mapped onto a number to allow fast and direct access to the edges via arrays. This means that each path connecting a node with the tree root describes a designated subsequence, which is simply the concatenation of all annotated characters of the edges. This subsequence is implicitly assigned to the node, which itself comprises a list of all sequences containing its assigned subsequence. To find all relevant sequences containing a particular subsequence, it suffices to walk along the tree choosing the edges according to the successive characters of the searched subsequence. The last node now contains the list of all relevant sequences.

**Figure 1:**
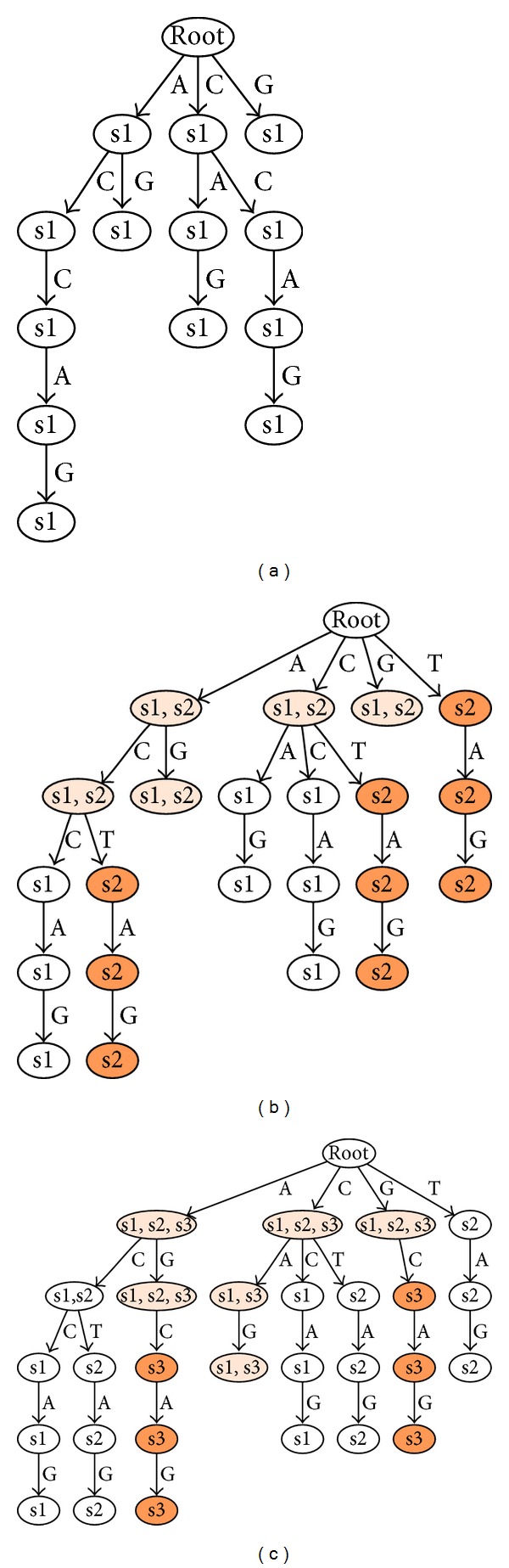
One example of the stepwise construction of the generalized suffix tree is shown, which later will be used within the search process. Parts (a), (b), and (c) of the graphic show the state of the tree after inserting the sequences ACCAG as s1, ACTAG as s2, and AGCAG as s3. Each edge is annotated by the corresponding letter of the underlying alphabet. The nodes themselves contain the list of sequence identifiers for all sequences containing the subsequence denoted by the path leading to the particular node. Starting from part b the coloring of the nodes indicates their status of update. Red colored nodes have been added in the latest construction step; orange nodes have been modified.

The tree is constructed by the repeated insertion of all sequences of the data set. As its model is not intended to map variable positions, all sequences containing variable characters are discarded as a first filtering step. The quality of today's sequencing technologies and appropriate preprocessing keeps the impact of the filtering insignificant. A single sequence is inserted into the tree by traversing the tree, beginning from the root node. The depth of this insertion traversal is limited by the maximum allowed motif length. If the next edge and connected node, which are chosen by the next character in the inserted sequence, do not exist during traversal, they are created and the procedure is continued. Each node that is traversed during the insertion process will have placed the sequence ID of the inserted sequence into its internal list. Duplicate entries in the nodes internal lists are avoided. As we are creating a suffix tree, not only the sequence itself but also all possible suffixes of the inserted sequence need to be processed in the same manner to complete the insertion of a single sequence. According to this principle all sequences of the data set are inserted consecutively as shown in the example of tree creation in three steps in Figure 1. The time and space complexities of tree creation are within *O*(*n* · *l* · *r*), where *n* is the number of sequences, *l* the sequence length, and *r* the maximal allowed motif length.

**Figure 2:**
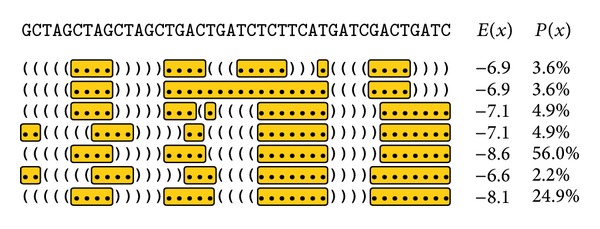
The selection and weighting of secondary structure information prior to tree creation is illustrated. The top row shows the sequence of interest. In the following rows, for each secondary structure predicted by the Vienna RNA tool RNAsubopt [23], a representation in dot-bracket notation is placed. Mutual matching pairs of brackets denote base pairs, whereas dots are standing for unpaired bases. Each structure *x* is annotated with an energy value *E*(*x*) and the calculated probability *P*(*x*) used for weighting. The nonpaired subsequences, which will be inserted into the tree, are highlighted yellow. It is obvious that some subsequences are inserted multiple times and others are overlapping, which necessitates the mentioned weighting.

In particular loop regions of nucleotide aptamers are likely to interact with target molecules [11]. As the unpaired nucleotides in loop regions do not take part in Watson-Crick or other kinds of nucleotide pairs, the related binding sites remain available for intermolecular chemical bonding. Loop regions should therefore be preferred when searching for common binding motifs. To adapt the presented strategy towards possible loop regions and potential binding motifs, the construction process of the tree was modified. To determine which parts of the sequences are placed on unpaired regions, the corresponding secondary structures need to be predicted. However, taking only into account the best predicted structure may lead to unintended findings, because predictions can only be trusted to the extension of their predictive performance. In the concrete binding situation many external impacts will influence the folding of the aptamer, so that the structure of the highest binding affinity does not necessarily correspond to the structure yielding minimal free energy. However, the latter is the objective in structure prediction algorithms. Thus, in the context of developing aptamer-target-binding models, RNA structure predictions have to be regarded with care and caution. Therefore a set of suboptimal structures is used as basis, which is predicted with the tool RNAsubopt of the Vienna RNA toolbox [32]. Hence the RNAsubopt application is primarily designed to be applied on RNA sequences; the prediction of DNA secondary structures requires a different energy parameterization [33, 34]. As the primer sequences are attached to the main aptamer sequence during the incubation phase, they are influencing its structural fold. Due to that the primer sequences need to be attached prior to predicting the aptamer secondary structures and neglected after prediction. For each of the predicted structures of each sequence, all loop subsequences are extracted and separately inserted into the tree. Loop regions that are contained in more than one suboptimal structure are now inserted multiple times. For a correct interpretation in the later pattern search, the inserted loop regions have to be weighted. The selection and weighting of unpaired structure elements is depicted in Figure 2. Besides a standard equal weighting of all structures, a special weighting according to the secondary structures annotated free energy values can be realized. Therefore a kind of probability for each sequence to be found in a natural mixture is calculated by the following formula using Boltzmann factors based on these energy values [35]:

(1) $$\begin{array}{c} P\left(x\in X\right)=\frac{1}{Z}\cdot e^{-1/\left(k_{B}\cdot T\right)\cdot \beta \cdot E(x)}\\ Z=\sum_{x\in X}e^{-1/\left(k_{B}\cdot T\right)\cdot \beta \cdot E(x)}. \end{array}$$

For each of the structures *x* of the ensemble *X*, an energy value *E*(*x*) needs to be available. The Boltzmann constant *k*_*B*_ and the absolute temperature *T* in kelvin are also required for calculation. The temperature value should be consistent with the settings used in secondary structure prediction. An additional parameter *β* allows customizing the characteristic of the weighting function. Larger values of *β* increase the up-weighting of better energy values; smaller values weaken the influence of the predicted free energy. A value of 0 for *β* comes to an equal weighting of all structures. The partition function *Z* can be seen as a normalization factor, so that the sum of all calculated probabilities will not exceed the limit 1.

Now the tree can be constructed either with or without using the information provided by the predicted secondary structures.

### 3.3. Motif Search Using Node Merging

The motif search algorithm shall be able to find motifs containing variable regions. As the underlying tree structure only models nonvariable strings, the variability needs to be realized within the search process. Therefore a new composite alphabet is created and used as basis for the following search. This composite alphabet contains all standard characters taken from the normal sequence alphabet and all possible combinations as special, variable characters. The composite alphabet can easily be restricted to only 2-letter or 3-letter combinations. With the help of this composite alphabet, the search now is able to cover variable motifs.

**Figure 3:**
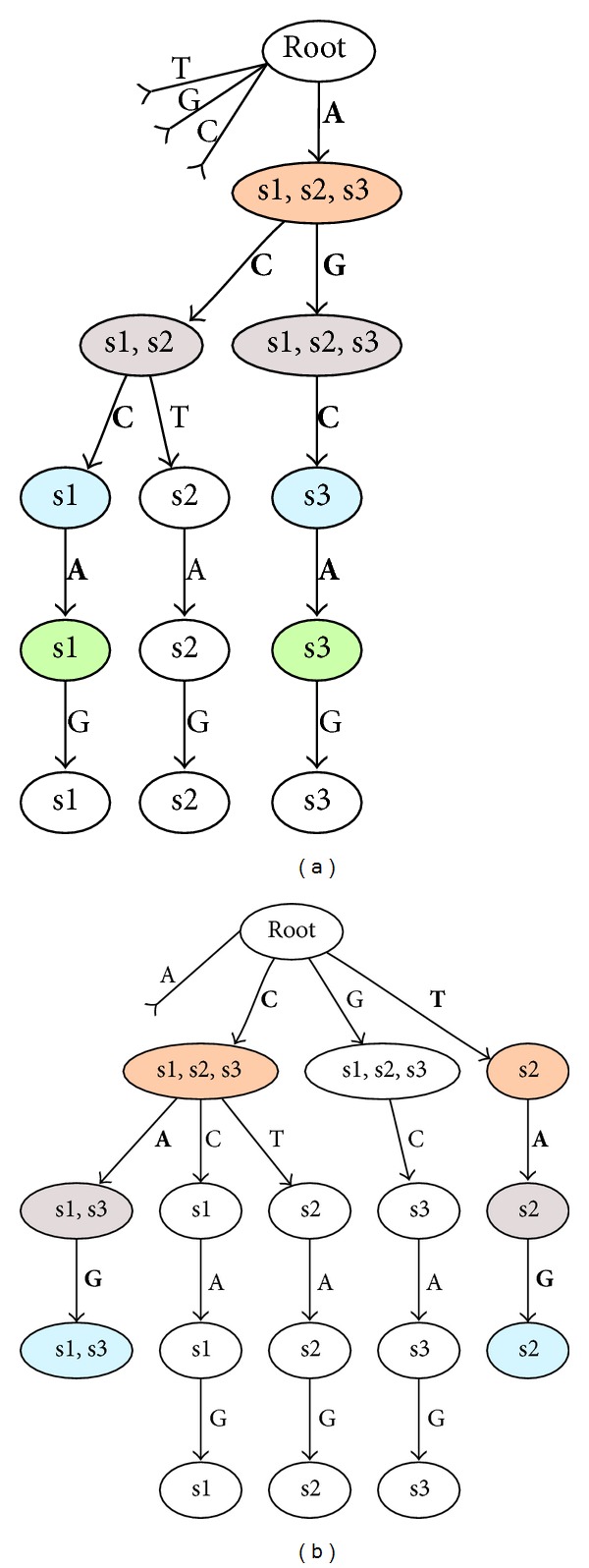
The search process within the suffix tree in two examples using variable motif positions is illustrated. In both examples parts of the tree have been omitted for visual perspicuity purposes. The basis is the tree, which was constructed in Figure 1. The changing colors from one row to another are for visual distinction of the consecutive node sets during search. Letters corresponding to the chosen edges are printed in bold font weight. In (a) the motif A[CG]CA is searched, which leads to a fork in the tree at step two. It is not necessary to traverse the tree down to the leaves. Merging the green nodes on the last marked row offers the list of search results (s1, s3). (b) searches for the motif [CT]A[GT]. In the last step there is no suitable edge found for the second allowed character T. Merging the cyan nodes on the last marked row offers the list of search results (s1, s2, s3).

Performing the full search with the help of a suffix tree allows a very fast substring search strategy for both, normal and variable substrings. This strategy uses the principle of progressive node merging, which is a depth-first search. Each search process starts with the empty string, which is represented by the root node of the tree. As we are using node sets, the starting node set only consists of the trees root node. For each possible following character the search is continued. The following character can also be a combination of more than one character of the original sequence alphabet, because a composite alphabet is used. To continue the search, a new set of nodes covering the next searched substring with all variabilities needs to be found. This is done by aggregating all subnodes of nodes contained in the current node set, whose edges correspond to the composite character currently processed. The obtained node set is now merged to retrieve a single list of sequences containing the pattern represented by the nodes. This principle is demonstrated on two simple examples in Figure 3. Since a single sequence may occur in multiple nodes, the merged list of sequences has to be cleaned from redundant entries. Instead of holding a sorted list or linearly searching for each sequence prior to inserting it, an index list helps to ignore doublets with minimal time overhead, only requiring the sequences to carry a serial number. Now the merged node holds all sequences containing the searched pattern. The search effort for a pattern with one additional character is therefore minimal, because neither an actual string search nor a full tree traversal needs to be accomplished for each step of the motif search. The space complexity is within *O*(*n*), where *n* is the number of sequences. However, due to the exhaustive search the maximum time complexity of the search is within *O*(|Σ*|^*r*^), where Σ* is the compound alphabet and *r* the maximal allowed motif length. The following termination criteria will reduce the required computational effort.

### 3.4. Termination Criteria

Two straightforward termination criteria are defined when starting the search procedure. The first criterion is the maximal motif length, which bounds the depth-first search at a specific depth. The second is the quantity of sequences containing the current motif. As a motif extended by one additional character must be equal or less frequent than the original, the search branch can be cut when the limit of quantity is reached.

For all further criteria, the motif actually contained in the found sequences needs to be constructed. Therefore all actual occurrences of the motif, which are located in the merged nodes internal list, are analyzed position by position. A result of this analysis is a position specific scoring matrix (PSSM), which now can be used for comparison and further calculation.

Another termination criterion is the formal integrity of a discovered motif. That means the exact match of the found motif with the motif actually contained in the sequences which is denoted by the PSSM. If these motifs do not match, because at some position of the actual motif an original character is missing, the branch can also be rejected, because the presence of another (namely, the actual) motif covering that branch is mandatory.

Some other restrictions are only applicable for motif filtering, but not for termination of the search branches. Besides the minimal motif length, the entropy based total information of single positions of a motif and the average total information of all positions of the motif can be mentioned here. As the entropy *H* of an event, in this case of the event described by the probability distribution of one position in the PSSM, is a measure of the uncertainty, its complement can be used as a measure of expressiveness. We have chosen the Shannon entropy *H* = −∑_*i*=0_^*N*^ *p*_*i*_ · log⁡_2_ *p*_*i*_ which uses *p*_*i*_ as the values for probability or relative frequency of the characters in one column of the PSSM and *N* as the original alphabets length. It has a maximum value of *H*_max⁡_ = log⁡_2_ *N*. The total information *E* is then simply the difference *E* = *H*_max⁡_ − *H*, which leads to values from 0 at uniform distribution to 2 for a nonvariable position [36].

However, a limitation of the total information values as described would result in the avoidance of possible gaps, which means positions of low total information. If they are desired to be found, defining another upper limit of total information to identify gaps, which are not validated by the standard total information criterion, will help. Motifs starting or ending with such a gap can be discarded without any consequence.

### 3.5. Aggregation of Motif Results

**Figure 4:**
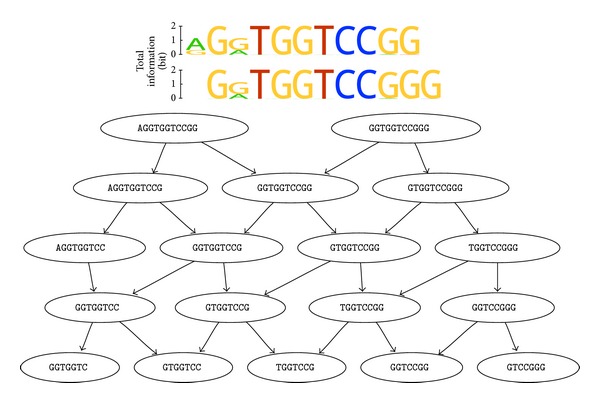
The result of the performed motif search is shown. In the lower part of the figure, the consensus sequences of the 18 discovered groups, which contain the actual motifs, are depicted. This is done in the form of a directed graph showing a substring relation. That means that a consensus sequence is connected to all other sequences, which are substrings of itself. The emerging hierarchy facilitates the understanding and selection of relevant finds. The upper part shows two concrete motifs in the form of weblogos, which have been picked one from each of the top consensus groups and then have been aligned to each other. The height of each column of the two motif weblogo representations corresponds to the motif positions total information according to the scale on the left side. The letters are then sized by their relative frequency within that motif position.

**Figure 5:**
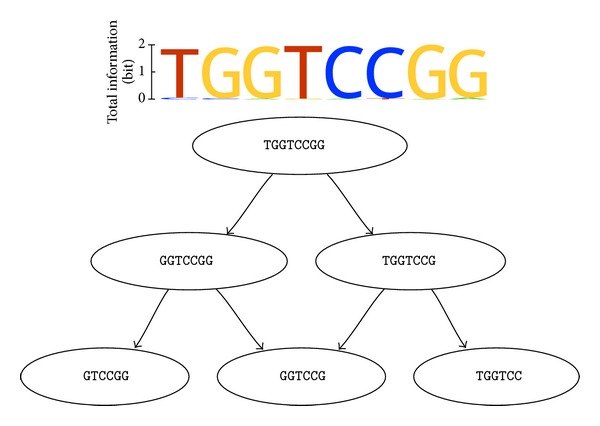
The result of the performed motif search using the secondary structure restrictions is shown. In the lower part of the figure, the consensus sequences of the 6 discovered groups, which contain the actual motifs, are depicted. This is done in the form of a directed graph as in Figure 4. The upper part shows one concrete motif in the form of a weblogo, which has been picked from the top consensus group. See Figure 4 for further explanation of the weblogos representation.

In consequence of the allowed variability and the used naive search strategy, a very large number of motifs will be eventually found, and thus the result of the algorithm will be difficult to manage. However, the resulting motif hits will naturally form a number of motif groups offering high mutual similarity, because the variability at each position leads to some kind of vacillation around a main motif. One possible solution to relieve the manageability is to group found patterns together by using an easy derivable consensus sequence of each pattern. A directed graph connecting a motif to other motifs, which are substrings of itself, is the preferred visual representation as seen in Figures 4 and 5.

## 4. Results

### 4.1. Normal Motif Search

For a motif search, the most frequent 1000 distinct sequences of the last round of the SELEX run have been chosen. The search was limited to motifs of length 7 to 11 and shall only show results with minimal total information of 1.8 bits, which occur in at least 95% of the approximately 233,000 concerned sequences. The variability was constrained by allowing only one or two original characters in each character of the composite alphabet.

The motif search results in approximately 150,000 motifs, which can be separated into 18 groups. The 18 groups are shown in Figure 4. The two longest consensus sequences of the groups are overlapping and thereby forming the motif (A)GGTGGTCCGG(G). The other 16 found groups show consensus sequences, which are subsequences of the two largest finds. The main focus shall therefore be laid on the two longest finds. Looking at the concrete formation of the motifs contained in these two groups shows that the only noticeable variability lies in positions 1 and 3 of the motifs. This yields the overall motif description of [AG]G[AG]TGGTCCGGG.

The sequence data set was also submitted to different motif search webservices. Only two of the tested services were able to handle the large data set. DREME returned 50 motifs offering 4600 to 40 matches within the given 5500 distinct input sequences [37]. The DRIMust online service resulted in a list of overrepresented k-mers and one motif hit [38]. The first motif hit reported by DREME as well as the top elements of the overrepresented k-mers provided by DRIMust corresponds to the motif found by this approach, whereas the DRIMust motif and later motif hits reported by DREME do not match to our result. The extended use of variability in combination with the exhaustive search strategy facilitates the finding of motifs that fit the natural variation more precisely. Due to this a very strict threshold could be applied to sequence coverage (95%) during the motif search.

### 4.2. Using Secondary Structure Information

In a second run, the secondary structure information was used to select only subsequences for motif search, which are likely to be located on loop regions of the structure. For that reason a suboptimal secondary structure prediction with an allowed energy delta of 1 kcal/moL was chosen. The absolute temperature *T* was set to 310 K and parameter *β* was set to 1. As this selection restricts the number and length of subsequences, which provide the basis for the motif search, using the same severe parameters as above will cause the search to reveal a reduced result focused on the loop regions.

With the altered base set the algorithm discovers approximately 125 motifs, which are aggregated into 6 consensus groups shown in Figure 5. The group with the longest consensus sequence is TGGTCCGG, which is a subsequence of the motif discovered without using secondary structure information. The other finds are subsequences of this motif. The circumstance that the motif discovered with structural restrictions is a subsequence of the one found without such restraints supposes that the found motif is relevant for binding to the target.

As we initially introduced a weighting based on the predicted free energy of the secondary structures, each found motif now contains a value describing a kind of propensity or probability for this motif to be found on loop regions of the aptamers structure. The longer the desired motif, the lower the expected propensity. So the longest motif TGGTCCGG is accompanied by a value of around 65%. The most common group TGGTCC in contrast ranges from values of 71% to 80% and is therefore probably assembled of unpaired nucleotides.

### 4.3. Validation

As a manual validation the 25 most frequently occurring sequences of the data set have been checked. After the aggregation of the sequences into six groups of mutual global similarity, the consensus sequences of these groups were inspected. All except one sequence did contain the motif [AG]G[AG]TGGTCC[GA]GG, where only a small percentage is responsible for the last variable position. The one remaining sequence does only contain the motif TGGTC[]GGG with one missing C in the middle of the motif. One aptamer containing the found motif has also been experimentally confirmed to bind to the target.

**Figure 6:**
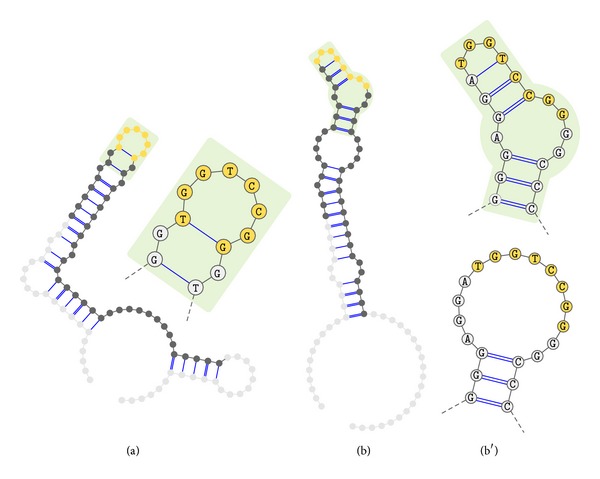
The result of mapping the found motif TGGTCCGG onto different predicted secondary structures of aptamers frequently occurring within the final SELEX round is shown. This is done using two examples. In both cases, based on the output of the VARNA [27] online tool, the optimally predicted secondary structure is schematically drawn with the following coloring. Light gray circles are nucleotides of the primer sequences, whereas dark gray and yellow circles are nucleotides of the actual aptamer. The latter are containing the searched motif. The area containing the motif is shaded in a light green tone and additionally presented in a separated detail view besides. In (a), the motif is exactly matching a hairpin loop. In (b) the motif is distributed over paired and unpaired nucleotides. A second detailed view (b′) shows the same part based on a suboptimal structure instead providing a larger loop as an only difference, which holds the motif.

For the top sequences of the groups determined above, secondary structures have been predicted separately to map the found motif onto the possible aptamer structures. The visualization of the structures was done with the online tool VARNA [39] and is presented in Figure 6. In some cases the optimal predicted structure contained the motif positioned on an unpaired region. Although the motif was positioned partially or even fully on paired regions in the other considered cases, a suboptimal structure with small energy difference existed, on which the motif was found nearly or fully on a structure element consisting of unpaired nucleotides. This finding can be attributed to the new methodology incorporating the predicted secondary structure information into the motif search process.

### 4.4. Library Generation

The SELEX experiment resulted in a final library with decreased diversity. Using the NGS data this decrease has been validated by calculating the diversity measures Simpson index and Shannon-Weaver index [40]. Corresponding to that diversity an enrichment of a number of aptamer sequences within the library can be observed. Besides a simple grouping of the sequences by global similarity, another approach, the motif search, was pursued. As a result of this performed motif search a short motif was revealed, which could be found in more than 95% of the investigated sequences. This motif is furthermore positioned on a loop region of suboptimally predicted secondary structures in the majority of the cases. This leads to the assumption that the motif TGGTCCGG is especially relevant for target binding, because loop regions offer unpaired nucleotides whose binding sites remain available for intermolecular chemical bonding.

As shown above, the motif corresponds to similar substructures within the different enriched aptamers, which may fit characteristically onto a specific binding site located on the target protein. This circumstance can be used to generate an enhanced SELEX starting library, which in turn will positively affect the progress of future SELEX runs on the same target molecule. As the discovered motif is described by a position specific scoring matrix, the natural divergence is captured and can be used when creating the new library. The motif itself represents a kind of indication for a preferred aptamer binding site; it is not a fully qualified predefinition of the optimal and exact binding aptamer. A SELEX library should therefore be enriched by the motif. One possibility is to create a small preliminary library highly enriched with that motif, which is modified and thereby inflated in the process of postrandomization. Another way would be a randomized sequence generation with the restriction, so that the resulting sequences have to contain a small number of possible variant instances of the desired motif. By this means, the highly complex conformation space of the aptamers is filled diversely with structures containing different configurations of the potential binding motif. This ensures that also conformational changes of the aptamers induced by the influence of the target molecule and other environmental impacts while binding are abstractly regarded in the libraries creation process. Following SELEX runs can eventually profit from the target-specific enhanced starting library, which was designed by using the additionally gathered NGS sequence data.

## 5. Discussion

In a narrow sense, the correct application of the described method would imply that for each SELEX run, which shall profit from the target specifically generated new libraries, another SELEX experiment has to be performed to gather the sequence data required for finding the relevant motifs. In the direct manner this can be used after a performed SELEX experiment offering only aptamers of relatively low affinity. If motifs can be determined, a following SELEX experiment with optimized library could be used to find aptamers with higher affinity in fewer rounds. Another application is the optimization of the SELEX procedure. In normal cases the diversity decreases slowly in the later rounds of the experiment. The strategy discussed in this paper could reduce the number of necessary SELEX runs by introducing a sequence analysis step. After the analysis the experiment will be continued with a motif-based enriched library to have better chances to capture higher affinity aptamers.

**Figure 7:**
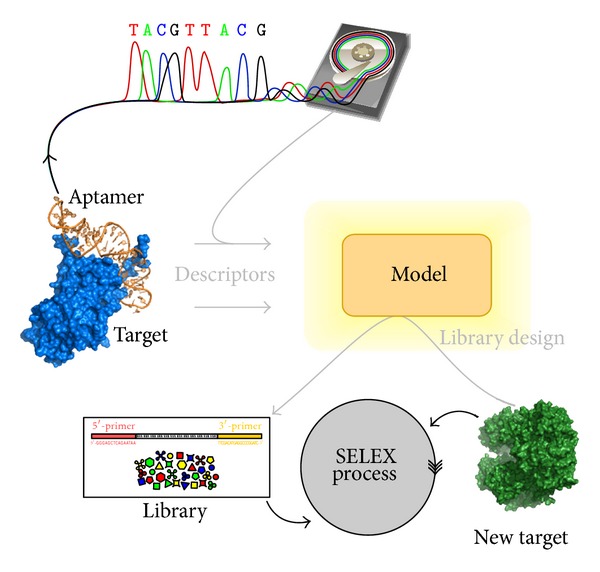
A schematic depiction of the longer term goal is shown. The left upper area illustrates the creation of an abstract model based on aptamer-target-binding information gained in the form of a multitude of descriptors for both, target and aptamer. The lower section illustrates the usage of the abstract model to generate a target-specific SELEX starting library only based on information about the desired new target molecule.

The found motifs can be seen as one descriptor for the target, because aptamers containing that motif are likely to bind to that intended target molecule. This can be a consequence of physiochemical preferences of the amino acids and nucleotides as well as concrete structural preferences of the motif. The shown method can be extended and thereby practically enhanced by making use of other available, mostly complex descriptors for the target and also for the aptamers. This starts with descriptors based on the pure sequence, for example, sequence alignments, consensus sequences, clusterings, and base or amino acid distributions, but is not limited to these. It is also possible to use available secondary or tertiary structures of the binding partners or to predict these structures, which then can be analyzed in terms of physical surface formation, electrostatics, buriedness, and availability of the different amino acids and nucleotides. It is also surmisable to use a docking simulation to validate or even identify potential binding sites, which then can be described in more detail. After describing both, target and aptamer, in an appropriate model by quantifiable descriptors, these values can be correlated in a new model abstractly describing the aptamer-target-binding relationship. Now the real practical benefit of the basic strategy becomes obvious. At this point, the model can significantly contribute to dry and wet lab investigations, since it is applicable to other, even structurally unknown target proteins, and can aid in gaining knowledge on the composition and architecture of binding aptamers only based on information about the desired target. The generation of target-specific SELEX starting libraries without the need of concrete performed previous experiments with the desired target as illustrated in Figure 7 would greatly improve the aptamer finding process in fields of biosensor development and medical treatment.

## 6. Conclusion

Performing NGS on SELEX experiments can yield benefits. Although this sequencing is not part of the standard SELEX procedure, the technique and following sequence analysis can help to find a better description of the developed enrichment within the library. In this paper the enrichment of a specific sequence motif has been shown by performing a motif search on the sequenced last round of a SELEX experiment. The high enrichment of sequences containing this motif and its likelihood to be located on unpaired regions of the aptamers indicate the motifs relevance for binding to the target protein. According to that the motif corresponds to a specific characteristic of the target. This kind of target description is only a first step towards an abstract model describing the aptamer-target-binding relationship, which then can be utilized to predict information on composition and architecture of binding aptamers. Based on this information SELEX starting libraries can be generated target-specific, which in turn will save time and financial expenses.
